# Using Natural Language Processing to Explore “Dry January” Posts on Twitter: Longitudinal Infodemiology Study

**DOI:** 10.2196/40160

**Published:** 2022-11-18

**Authors:** Alex M Russell, Danny Valdez, Shawn C Chiang, Ben N Montemayor, Adam E Barry, Hsien-Chang Lin, Philip M Massey

**Affiliations:** 1 Center for Public Health and Technology Department of Health, Human Performance, and Recreation University of Arkansas Fayetteville, AR United States; 2 Department of Applied Health Science School of Public Health Indiana University Bloomington, IN United States; 3 Department of Health and Kinesiology Texas A&M University College Station, TX United States

**Keywords:** alcohol, drinking, social media, Twitter, Dry January, infodemiology, infoveillance, natural language processing

## Abstract

**Background:**

Dry January, a temporary alcohol abstinence campaign, encourages individuals to reflect on their relationship with alcohol by temporarily abstaining from consumption during the month of January. Though Dry January has become a global phenomenon, there has been limited investigation into Dry January participants’ experiences. One means through which to gain insights into individuals’ Dry January-related experiences is by leveraging large-scale social media data (eg, Twitter chatter) to explore and characterize public discourse concerning Dry January.

**Objective:**

We sought to answer the following questions: (1) What themes are present within a corpus of tweets about Dry January, and is there consistency in the language used to discuss Dry January across multiple years of tweets (2020-2022)? (2) Do unique themes or patterns emerge in Dry January 2021 tweets after the onset of the COVID-19 pandemic? and (3) What is the association with tweet composition (ie, sentiment and human-authored vs bot-authored) and engagement with Dry January tweets?

**Methods:**

We applied natural language processing techniques to a large sample of tweets (n=222,917) containing the term “dry january” or “dryjanuary” posted from December 15 to February 15 across three separate years of participation (2020-2022). Term frequency inverse document frequency, k-means clustering, and principal component analysis were used for data visualization to identify the optimal number of clusters per year. Once data were visualized, we ran interpretation models to afford within-year (or within-cluster) comparisons. Latent Dirichlet allocation topic modeling was used to examine content within each cluster per given year. Valence Aware Dictionary and Sentiment Reasoner sentiment analysis was used to examine affect per cluster per year. The Botometer automated account check was used to determine average bot score per cluster per year. Last, to assess user engagement with Dry January content, we took the average number of likes and retweets per cluster and ran correlations with other outcome variables of interest.

**Results:**

We observed several similar topics per year (eg, Dry January resources, Dry January health benefits, updates related to Dry January progress), suggesting relative consistency in Dry January content over time. Although there was overlap in themes across multiple years of tweets, unique themes related to individuals’ experiences with alcohol during the midst of the COVID-19 global pandemic were detected in the corpus of tweets from 2021. Also, tweet composition was associated with engagement, including number of likes, retweets, and quote-tweets per post. Bot-dominant clusters had fewer likes, retweets, or quote tweets compared with human-authored clusters.

**Conclusions:**

The findings underscore the utility for using large-scale social media, such as discussions on Twitter, to study drinking reduction attempts and to monitor the ongoing dynamic needs of persons contemplating, preparing for, or actively pursuing attempts to quit or cut down on their drinking.

## Introduction

### Background

“Dry January”—a public health campaign aimed at encouraging individuals to reflect on their relationship with alcohol by temporarily abstaining from consumption during the month of January—originated in the United Kingdom in 2013 [1,2]. Those who register to participate in the month-long challenge via the Alcohol Change UK website are provided added accountability and support through access to interactive online resources (eg, TryDry mobile application) and health communication messaging highlighting the benefits of temporary alcohol abstinence (eg, emails and social media messaging about financial health, physical health, and mental health benefits) [3]. Dry January is theorized to confer benefits to participants via social contagion, which suggests widespread changes in health beliefs and behaviors are more likely to occur when a supportive community or subgroup of people endorse similar motivations and goals [4-6].

Prior research evaluating the characteristics of Dry January participants and the efficacy for the campaign in terms of reducing alcohol consumption and enhancing quality of life indicators has primarily focused on official Dry January registrants (ie, those who reside in the United Kingdom and officially registered for the challenge on the Alcohol Change UK website) [7-9]. Most of these studies have demonstrated that official participation in the temporary abstinence initiative is associated with numerous short- and long-term benefits, including reductions in alcohol consumption, increases in alcohol-refusal skills, saving money, improved sleep, increased energy, weight loss, and enhanced psychological well-being [5,7-9]. However, Case et al [10] found that increased participation in Dry January in England between 2015 and 2018 was not associated with population-level reductions in alcohol consumption over the 4-year period.

One potential explanation for these mixed findings could be that, although the number of officially registered Dry January participants in the United Kingdom has risen from 4000 in 2013 to 130,000 in 2021 [1], this represents only a small minority of the public who are informally participating in the temporary alcohol abstinence initiative (an estimated 6.5 million Britons reported planning to give up alcohol during the month of January in 2021) [11]. Additionally, the reach of the Dry January campaign has extended beyond the United Kingdom and has become a global cultural phenomenon with millions of informal participants worldwide [12]. For example, an estimated 15% to 19% of American adults reported going alcohol-free during January 2022 [13,14]. This has coincided with increasing news media attention [15,16], social media engagement, and Dry January–related alcohol industry promotional efforts (eg, marketing of nonalcoholic alternatives) [17]. For the millions of individuals who *unofficially* participate in alcohol abstinence during the month of January, there remains a paucity of investigations and a need to better understand their experiences in attempting to abstain from alcohol during the month of January. One such means through which to gain insights into individuals’ Dry January-related experiences is by leveraging large-scale social media data (eg, Twitter chatter) to explore and characterize public discourse concerning Dry January. 

### Infodemiology

Infodemiology (the epidemiology of online information, such as using search result data or social media posts to inform public health and policy) and infoveillance (longitudinal tracking of online information for surveillance purposes) are emerging fields [18-21]. The last decade has witnessed a proliferation in Twitter and other social media platform usage, and many individuals rely on these platforms for health information [22-24]. Along these lines, infodemiology methods have been used to systematically monitor public sentiment and characterize communication concerning various health topics using publicly available social media data, such as Twitter posts [21]. Though not intended to replace, but rather complement, more traditional methods, infodemiology offers several advantages, including the ease and rapidity with which data can be collected, allowing for the ability to detect changes in public attention and attitudes in real time [18-20]. Previous studies leveraging Twitter as a data source have provided insights into a variety of health topics, including alcohol-related behaviors [25-28], tobacco use and cessation [29-32], drug use [33,34], mental health [35,36], vaccination [37,38], and the spread of health-related misinformation [39]. Moreover, Twitter has been used as a real-time surveillance tool to monitor reactions to public health prevention campaigns [40] and public policy changes [41,42], providing timely information to public health researchers, practitioners, and policy makers.

### Alcohol Use Infodemiology on Twitter

A growing number of studies have explored alcohol-related, user-generated content posted on Twitter [25-28]. For instance, Cavazos-Rehg et al [25] was among the first to characterize a large sample of alcohol-related tweets, finding that the vast majority of such tweets expressed positive sentiment toward alcohol and frequently glamorized heavy drinking, while rarely portraying any alcohol-related negative consequences. Other studies have examined tweets concerning alcohol-related blackouts [26,28,43]; increases in alcohol-related blackout tweets in early 2020 were in line with population-level increases in alcohol consumption observed during the COVID-19 pandemic [28]. Weitzman et al [44] compared state-level alcohol use–related Twitter posts and Google Trends search data with 3 years of national epidemiological survey data, providing support for using search activity and social media data to complement epidemiological approaches to monitor alcohol use and inform prevention efforts. However, there has been a dearth of infodemiology studies focused on efforts to quit or cut down on drinking, such as drinking reduction attempts associated with the Dry January temporary alcohol abstinence campaign [8,9].

### This Study

The purpose of this study was to identify and describe a corpus of Dry January–related tweets authored by the public and social bots across 3 years of participation (2020-2022) and to evaluate whether there were changes in themes and sentiment from year to year in response to the COVID-19 pandemic. We sought to compare conversational themes over time to demonstrate the potential use for social media platforms—such as Twitter—to be used to study drinking reduction attempts and to monitor the ongoing dynamic needs of persons actively involved in or thinking about attempts to quit or cut down on drinking. To achieve this objective, we applied natural language processing (NLP) techniques to a large sample of Twitter data (n=222,917), spanning 3 distinct years (2020-2022), to answer the following research questions (RQs):

(RQ1) What themes are present within a corpus of tweets about Dry January, and is there consistency in the language used to discuss Dry January across multiple years of tweets (2020-2022)?
(RQ2) Do unique themes or patterns emerge in Dry January 2021 tweets after the onset of the COVID-19 pandemic?
(RQ3) What is the association between tweet composition (ie, sentiment and human-authored vs bot-authored) and engagement with Dry January tweets?

## Methods

### Data Collection

Tweets associated with this study, including metadata (eg, number of likes, retweets, replies) were extracted using the Twitter application programming interface (API) v2 and Python 3.9. After obtaining approval for access to the Academic Research product track of Twitter’s API v2, we identified and extracted all tweets containing the term “dry january” or “dryjanuary” posted from December 15 to February 15 across 3 separate years of participation (12/15/2019 to 02/15/2020, 12/15/2020 to 02/15/2021, and 12/15/2021 to 02/15/2022). Capturing the 2 weeks prior to and after the month of January allowed us to analyze conversations related to anticipation of Dry January, as well as those reflecting on completed Dry January attempts (whether successful or unsuccessful). We excluded all retweets, defined as the same tweet appearing multiple times in the corpus, and non-English tweets, defined as any tweets not originally written in the English language. Note, eliminating duplicate tweets and non-English tweets was done to enhance the interpretability of the NLP analyses undertaken herein [45]. Overall, 70,215 tweets were extracted from 12/15/2019 to 2/15/2020, 86,378 tweets from 12/15/2020 to 2/15/2021, and 66,324 tweets from 12/15/2021 to 2/15/2022, resulting in a final sample of 222,917 tweets. All tweets collected for this study, inclusive of nonpersonally identifiable metadata, were saved into a secure repository only accessible by the research team, strictly conforming to standards for ethical data use and online privacy.

### Ethical Considerations

Research procedures were deemed exempt by the appropriate institutional review board prior to data collection from Twitter.

### Analyses

Our research questions were exploratory in nature. As such, we strategically selected several classes of computational informatics methods designed to extract overall themes in the corpus and project relative similarity and dissimilarity across themes. These methods can be classified into those used for data visualization (term frequency inverse document frequency [TF-IDF], k-means clustering, and principal component analysis [PCA]) and for data interpretation (latent Dirichlet allocation [LDA] topic models, Valence Aware Dictionary and Sentiment Reasoner [VADER] sentiment analysis, and Botometer automated account check).

#### Data Visualization (Research Questions 1 and 2)

##### Term Frequency Inverse Document Frequency

TF-IDF refers to an information retrieval technique used to transform text data into numeric data [46,47]. Specifically, the TF-IDF algorithm creates weights for each word in a corpus, such that weights implicate (1) how important a word is in a singular tweet relative to (2) the number of times the same word was used in the entirety of the corpus. Weights per term can be interpreted as greater values equating higher word importance and lower values equating lower term importance. These weights are then transposed into a sparse matrix for further analysis.

##### K-means Clustering

K-means clustering is an unsupervised machine learning tool used to group text content into themes, or clusters. This analysis relies on the sparse matrix created by the TF-IDF calculations to categorize tweets into one of the k-clusters. The optimal number of k clusters is identified by calculating the sums of squared differences for a range of possible clusters (ie, 1 cluster to 10 clusters). The sums of squared differences for a range of k clusters are plotted along an elbow scree plot, where breaks in a plotted line indicate a possible clusters solution. For more information on k-means clustering, please see Na et al [48].

##### PCA

PCA, a commonly used analysis in exploratory factor analysis, is a dimensionality technique used to reduce the complexity, or components, of data while still maintaining the integrity of the data [49,50]. For text mining analysis, all words assigned weights by TF-IDF that have been assigned into one of the k-clusters are reduced into simple X and Y coordinates. These coordinates are transposed onto a vector map and color coded along the predetermined optimal k-clusters. For this analysis, we examined data shape, which simply refers to the way in which data are presented on a vector map.

#### Data Interpretation (Research Questions 2 and 3)

##### LDA Topic Models

LDA refers to an unsupervised NLP method that uses probabilistic inferencing to identify latent topics within a corpus of similar content. LDA is widely acknowledged as the most effective and precise topic modeling algorithm and has been widely applied for a variety of research areas and social issues [51,52].

##### VADER

VADER is a rule-based sentiment analysis attuned to social media vernacular [53,54]. VADER specifically examines the polarity of words in each tweet by feeding text data through a lexicon that is precoded with values for all positive and negative words in the English language. VADER scores can range from –.99 to .99. High values typically denote higher affect, or greater positivity, and lower values typically denote lower affect, or greater negativity.

##### Botometer

Botometer is a proprietary algorithm developed by the Indiana University Network Science Institute [55]. Botometer is widely used to determine if content in a tweet originates from an account that is principally human-authored or principally bot-authored. Users can leverage the Botometer API and search for specific user IDs or usernames and immediately receive a score from .01 to .99. Lower scores indicate that the account likely belongs to a human; higher scores, typically above .70, indicate that the account likely belongs to an automated bot. Note that, due to limitations with the Botometer API, we were only able to subsample 500 posts per cluster per year as a rough approximation of bot activity. Our decision to use a general .70 cutoff as a delineator between likely bot and likely human account is supported by Botometer validation literature and other studies leveraging Botometer for bot detection and removal [56,57].

#### Simple Inductive Coding and Validation (Research Questions 1, 2, and 3)

Although NLP methods can analyze language data en masse, a computer cannot ascribe meaning to themes derived from such analyses nor detect certain facets of human speech such as sarcasm [51]. As such, we invoked a simple inductive coding procedure in which 3 authors affiliated with this study independently reviewed approximately 50 posts per cluster per year. Authors were asked to describe the cluster in 3 or 4 words, and upon completion, the authors met to discuss overlap and differences. Key questions asked of the authors were to determine the overall content of each cluster, whether clusters were serious or humorous (ie, sarcasm), and whether the cluster seemed to promote a Dry January–related product. For humorous or sarcastic posts, we specifically looked for indicators, such as the presence of emojis, references to jokes, or exaggerated claims styled for likes. In circumstances in which unanimous consensus could not be reached, we repeated this process with 50 more randomly selected tweets until agreement was met. This process is generally deemed sufficient when dealing with mixed methods topic models on large-scale documents [58], though more research on uniform mixed methods topic modeling guidelines is needed.

### Procedure

**Figure 1:**
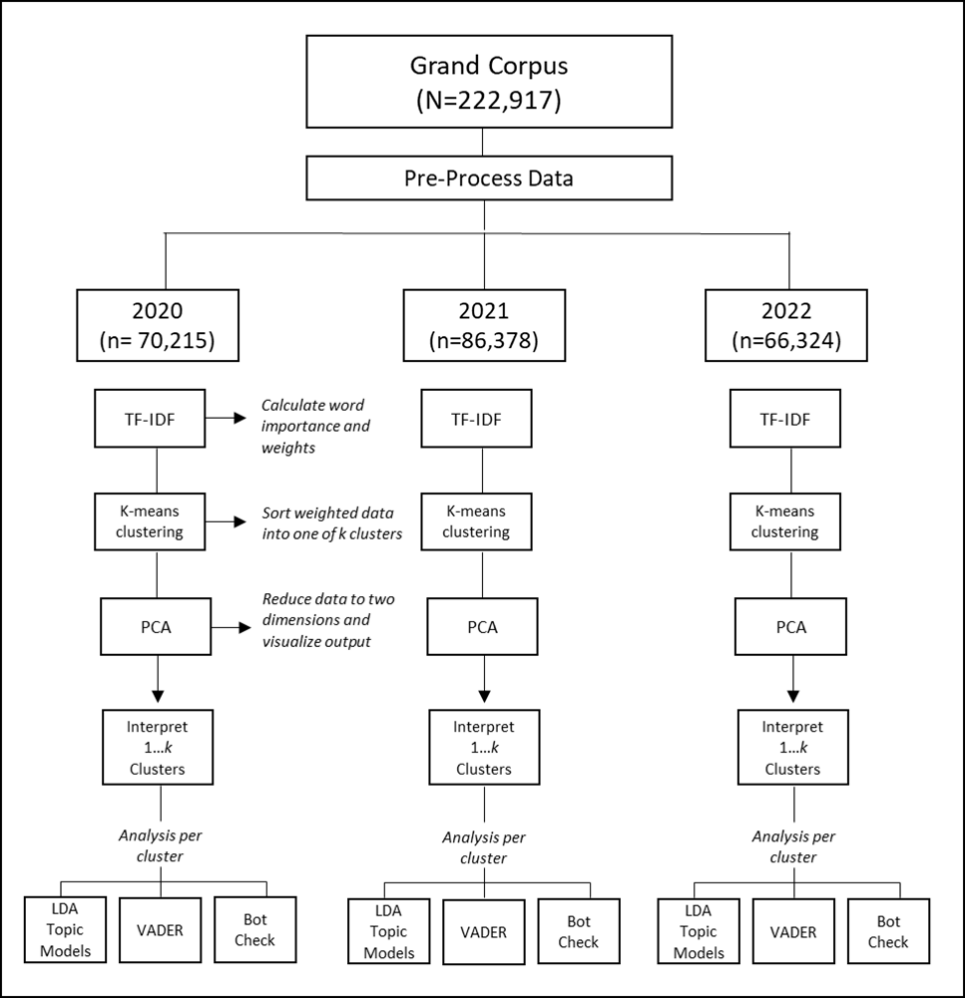
Study workflow detailing visualization and interpretation analyses per year. LDA: latent Dirichlet allocation; PCA: principal component analysis; TF-IDF: term frequency inverse document frequency; VADER: Valence Aware Dictionary and Sentiment Reasoner.

Our workflow is depicted in Figure 1. To prepare data for analysis, we initiated a series of preprocessing steps, including removing numbers, punctuation, and parts of speech that would detract from the readability of our models, including articles, prepositions, and contractions. Once all data were processed and cleaned, we divided our grand corpus into yearly iterations to afford content comparisons between years (RQ1). We ran a TF-IDF across every year (ie, 2020, 2021, and 2022), then used k-means clustering with elbow scree plots to identify the optimal number of clusters per year. We then applied a PCA to visualize our 2020, 2021, and 2022 data along a vector map. Once data were visualized, we ran interpretation models to afford within-year (or within-cluster) comparisons, including to determine the extent that a natural experiment, such as the COVID-19 pandemic, affected yearly Dry January–related content (RQ2). For example, we used LDA to examine content within each cluster per given year. We used VADER to examine affect per cluster per year. We used the Botometer to determine average bot score per cluster per year. Last, to assess user engagement with Dry January content (RQ3), we took the average number of likes and retweets per cluster and ran correlations with other outcome variables of interest including VADER and Botometer scores.

## Results

### RQ1. What Themes Are Present Within a Corpus of Tweets About Dry January, and Is There Consistency in the Language Used to Discuss Dry January Across Multiple Years of Tweets (2020-2022)?

**Figure 2:**
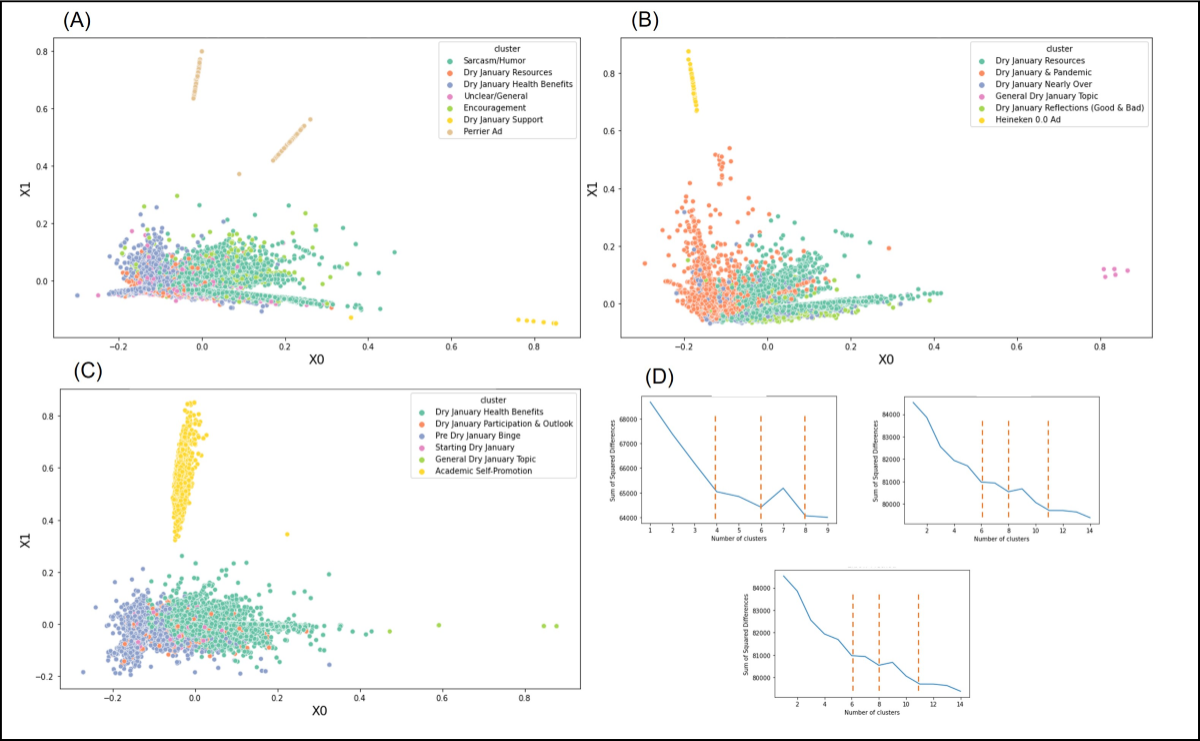
Composite figure with principal component analysis (PCA) visualization by year with model fit: (A) 2020 Dry January Twitter dialogue, (B) 2021 Dry January Twitter dialogue, (C) 2022 Dry January Twitter dialogue, (D) elbow method graphs.

First, we observed general consistency in topics over time. We used 2 measures to determine consistency of topics: (1) data shape (from the PCA) and (2) overlap in yearly topics (or repeating topics across each year of analysis). Figure 2 provides a visualization of our data per year and model fit summaries; Table 1 similarly provides general information for each year of data collection, topics per year and associated names, the number of tweets per cluster, engagement variables, and other indicators.

**Table 1:** Content cluster themes and associated summary statistics (n=222,917).

Year and topic			Results, n (%)		VADER^a^, mean^b^		Retweets, mean^c^		Likes, mean^c^		Quotes, mean^c^		Botometer score^d^
**2020 (n=70,215)**													
	Sarcasm/humor	38,242 (54.5)		0.16		0.82		9.10		0.12		0.37	
	DJ^e^ health benefits	5804 (8.3)		0.37		1.17		5.39		0.21		0.52	
	Perrier ad	1320 (1.9)		–0.93		0.00		0.12		0.01		0.88	
	Unclear/general	1458 (2.1)		0.03		0.32		4.28		0.07		0.37	
	DJ progress	3372 (4.8)		0.24		0.85		9.04		0.10		0.48	
	Perrier ad II	1334 (1.9)		0.93		0.00		0.13		0.01		0.88	
	DJ resources	16,390 (24.1)		0.36		0.77		4.18		0.10		0.44	
	Support & engagement	1755 (2.5)		0.29		0.50		7.80		0.08		0.39	
	Entire 2020 data set	N/A^f^		0.18		0.55		5.01		0.01		0.54	
**2021 (n=86,378)**													
	DJ nearly over	6190 (7.2)		0.2		0.72		12.39		0.17		0.49	
	Heineken 0.0. ad	953 (1.1)		0.61		0.007		0.07		0.003		0.9	
	DJ reflections	56,823 (65.8)		0.14		0.78		13.76		0.14		0.49	
	DJ resources	17,374 (20.1)		0.35		0.76		8.16		0.18		0.55	
	DJ & pandemic	3305 (3.8)		0.19		0.455		13.98		0.11		0.47	
	DJ general topic	1733 (2.0)		0.02		2.8		29.32		0.27		0.44	
	Entire 2021 data set	N/A		0.25		0.92		12.95		0.15		0.56	
**2022 (n=66,324)**													
	Starting DJ	2242 (3.4)		0.24		1.03		16.81		0.27		0.5	
	Academic self-promotion	1254 (1.9)		0.533		0.02		0.04		0.005		0.82	
	DJ health benefits	42,894 (64.7)		0.17		0.88		14.03		0.13		0.52	
	Pre-DJ binge drinking	15,183 (22.9)		0.37		0.7		5.85		0.09		0.67	
	General DJ topic	1447 (2.2)		0.03		0.4		7.97		0.07		0.52	
	DJ participation & outlook	3304 (5.0)		0.23		0.79		13.38		0.11		0.49	
	Entire 2022 data set	N/A		0.26		0.64		9.6		0.11		0.59	
Total			N/A		0.23		0.70		9.19		0.09		0.56

^a^VADER: Valence Aware Dictionary and Sentiment Reasoner.

^b^Mean scores were derived from scores ranging from –.99 (high negative affect) to .99 (high positive affect).

^c^A score of 1 indicates 1 retweet, like, or quote.

^d^Botometer scores range from .01 (low bot account likelihood) to .90 (high bot account likelihood).

^e^DJ: Dry January.

^f^Not applicable.

Using a coding procedure outlined in the previous sections, 3 authors affiliated with this study manually named each cluster using a series of representative tweets. Language in representative tweets posted by individual users subsequently included as exemplar tweets was slightly modified to capture original sentiment while preserving anonymity. Per each year, we observed several similar topics that suggest relative consistency in Dry January content over time. These topics include: (1) a general Dry January topic (eg, *“*Dry January yes, or no?”), (2) Dry January resources (eg, “Have you considered our app to help you maintain your #DryJanuary Goals?”), (3) Dry January health benefits (eg, “Here’s what one alcohol-free month can do for your mind and body”), and (4) updates (positive and negative) related to Dry January progress (eg, “Well, I only lasted a week of Dry January before I drank!”). In 2 of the 3 years included for analysis, we also observed corporate ads targeting Dry January participants, though similar ads were not apparent in 2022.

To support that yearly Dry January content was consistent, we also examined data shape (Figure 2). Indeed, our combined k-means and PCA approach demonstrates relative similarity and dissimilarity of clusters for each year of analysis. Clusters that are proximal contain similar content; clusters that are distal indicate dissimilar content. Though we acknowledge certain variation across each year, the data shape was relatively similar, which may indicate limited change in content over time. For example, in each year included for analysis, we observed 2 dominant clusters and several smaller clusters dispersed throughout the diagram. Additionally, for each year, we consistently observed at least 2 topics that were far removed and disconnected from the rest of the diagram. Topics, or clusters, that do not overlap with other clusters suggest pockets of conversation that are related to, but not necessarily embedded, within the larger conversation. A secondary explanation for consistent data shape may also be the cohesive theme of the grand corpus or subcorpora (ie, alcohol abstention during the month of January).

### RQ2. Do Unique Themes or Patterns Emerge in Dry January 2021 Tweets After the Onset of the COVID-19 Pandemic?

Our findings also indicate that Dry January was affected by emerging news cycles, most notably the COVID-19 pandemic. In the 2020 subcorpora, for example, we did not observe any tweets related to COVID-19, which would not become prevalent in the United States and Europe until March the same year. However, in the following year, we observed 1 cluster containing humorous content about Dry January’s cancellation due to the ongoing global pandemic (eg,”Bro, how can we do Dry January during a pandemic?” and “#DryJanuary is officially CANCELLED”). We also observed a small portion of tweets related to the January 6, 2021, US Capitol insurrection, though this content was less prevalent than COVID-19–related tweets. We did not observe a similar cluster related to COVID-19, or similarly disruptive news cycles, during 2022. Yearly news cycle changes may also explain variation in yearly data shape.

### RQ3. How Does Tweet Composition (ie, Sentiment and Human-Authored vs Bot-Authored) Affect Engagement With Dry January Tweets?

Tweet composition was associated with engagement, including number of likes, retweets, and quote-tweets per post. We used the Botometer and VADER sentiment analysis to test (1) whether bot-authored and human-authored posts had observed differences in engagement and (2) whether sentiment, which is calculated using the VADER lexicon, similarly affected tweet engagement.

For each year included in our analysis, we observed at least one bot-dominant cluster or an otherwise automated account that posts prewritten content. Per year, bot-dominant clusters were typically comprised of ads, such as Perrier Water and Heineken 0.0 beer, and to a smaller extent, paid or free resources to promote Dry January adherence. Bot-dominant clusters also had fewer likes, retweets, or quote tweets compared with human-authored clusters. Similarly, bot-dominant clusters also had the highest observed positive affect, or greatest amount of positivity per post (eg, “Ready to crush Dry January...with Perrier in your hands you are going to #MakeDryFly!!”). By contrast, human-authored accounts typically had greater engagement and contained lower affect, or greater amount of negativity (eg, “Bro I’m gonna DIE if I have to do another week of Dry January. LOL”). We note that lower affect may reflect sarcasm, though more research on this area is needed.

## Discussion

### Principal Findings and Implications

Our study characterized online content about Dry January, assessing trends, themes, and general attitudes toward the challenge. We used NLP tools to analyze and visualize a yearly series of tweets related to Dry January over the course of 3 years of participation. Our findings highlight that there is consistency in discussion themes about Dry January across multiple years of tweets, yet we were still able to detect unique themes that emerged in 2021 in response to the COVID-19 global pandemic. Additionally, tweet composition, or whether a tweet was bot-authored or human-authored and the sentiment of the tweet, was associated with user engagement (number of likes, retweets, and quote-tweets).

In the content cluster analysis of the corpus of Dry January tweets, several common themes emerged across multiple years of Dry January participation. For example, the promotion of Dry January resources—such as blogs with tips for help with sustaining Dry January efforts, mobile applications facilitating additional support and accountability, and recipes for nonalcoholic “mocktails”—was a consistent theme each year. Additionally, we observed a cluster associated with Dry January health benefits (eg, drinking reductions, weight loss, healthier dietary choices, reflecting on relationship with alcohol). These findings are consistent with prior work on Dry January that similarly highlighted reductions in alcohol consumption and weight loss as Dry January benefits, in addition to increases in alcohol refusal skills, saving money, improved sleep, increased energy, and enhanced psychological well-being [5,7-9]. Finally, a topic related to sharing about Dry January progress emerged across multiple years of data (eg, no desire to participate in Dry January, intention to participate in Dry January, failed attempts to abstain during Dry January, successful ongoing attempts, successful completion of Dry January). Although some tweets in this cluster referenced successful Dry January experiences and positive associations with these experiences, a large number of these tweets used humor and sarcasm to make light of Dry January participation and voiced an overall lack of desire to participate in the temporary abstinence initiative. This finding is in line with prior work examining alcohol-related content on social media platforms, such as Twitter and TikTok [25,26,59]; the vast majority of alcohol-related posts on these social media platforms portray drinking in a positive manner and often depict hazardous drinking behaviors, such as intoxication and blacking out, in a favorable manner. Similarly, alcohol-related negative consequences are rarely portrayed in alcohol-related social media posts, and when such portrayals are present, they are often depicted in a humorous manner that serves to downplay the severity of alcohol-related problems [25,59].

Content cluster analysis also detected unique themes related to Dry January across years, most notably a cluster of tweets related to Dry January participation in the context of the ongoing COVID-19 global pandemic during January 2021. Many of these tweets referenced individuals experiencing increased difficulty or a lack of desire to participate in Dry January in the context of the pandemic and social distancing restrictions and increased psychological stressors. Yet, others made reference to having an easier time abstaining during January due to the lack of access to social drinking activities. Humor was commonly used to make light of Dry January in the context of the pandemic. Subthemes within this cluster of tweets were consistent with prior research on alcohol consumption during the peak of the pandemic [60,61]. In addition to millions of COVID-19–related deaths, the COVID-19 pandemic has been associated with increased psychological stressors due to social isolation and higher unemployment rates, among numerous other factors [60,61]. Many have coped with COVID-19 pandemic stressors in the form of self-medication by increasing alcohol consumption [60,61]. Real-time infoveillance of social media posts may prove a valuable means through which to complement health behavior surveillance efforts and to detect public discourse and communication about unique health needs in response to big events, such as coping with the increased psychological stressors associated with the COVID-19 pandemic and how this may negatively impact efforts to quit or cut down on drinking [62].

Finally, we found that tweet composition, most namely whether a tweet was bot-authored versus human-authored affected online engagement with posts. That is to say, bot-dominant clusters (eg, Perrier and Heineken 0.0 promotional efforts) had fewer likes, retweets, and quote-tweets compared to primarily human-authored clusters. This finding has implications for public health messaging and intervention on social media platforms. Although there may be public health benefits from the development and facilitation of social bot-oriented online interventions [63], investigation is warranted into how best to tailor such intervention efforts to enhance engagement, as it appears many individuals in this study largely ignore posts from automated accounts with prewritten content. That said, without knowing the goals or intended outcomes of the bot creators (ie, generating content vs sharing content or raising awareness vs generating engagement), we are unable to determine the effectiveness of social bot presence in Dry January content on Twitter. Our findings do support the presence of social bots and their potential to create, share, and engage with online content.

### Limitations

This work is subject to limitations we hope to address in future work. First, although a combined k-means and PCA approach has been extensively validated as an effective way to analyze and visualize abundant social media content, this approach is exploratory and relies on unsupervised algorithms to arrive at findings. As such, there is a possibility that a small proportion of tweets may have been miscategorized by the algorithms. Second, given financial limitations with the Botometer API, we were unable to calculate Botometer scores for all tweets included in the analysis. Instead, we relied on generalizing the Botometer scores from a random subsample of 500 tweets per cluster. It is possible that a full Botometer analysis with the entire sample would alter our findings slightly, particularly for larger clusters comprised of tens of thousands of tweets; however, significant cost barriers associated with the Botometer API prohibited access to a full analysis of tweets. Finally, we also acknowledge that we did not perform a full qualitative analysis with these data. Although we maintain our blinded coding procedure to name clusters was sufficient to determine cluster names, there is also a possibility that a full review of all tweets in a given cluster would yield marginally different cluster names. Through the limitations outlined, we offer several compelling research opportunities to continue this study. For example, a comparative study contrasting our findings from those generated using supervised NLP algorithms, for example the Sentence Bidirectional Encoder from Transformers (S-BERT), could help validate our findings particularly if there is strong overlap across analyses.

### Conclusions

We explored themes within and across 3 separate years of Twitter posts about the Dry January temporary alcohol abstinence challenge. Although there was overlap in themes across multiple years of tweets, unique themes related to individuals’ experiences with alcohol during the midst of the COVID-19 global pandemic were detected in the corpus of tweets from 2021. Findings underscore the utility for using large-scale social media, such as discussions on Twitter, to study drinking reduction attempts and to monitor the ongoing dynamic needs of persons contemplating, preparing for, or actively pursuing attempts to quit or cut down on their drinking.

## Abbreviations

API: application programming interface
DJ: Dry January
LDA: latent Dirichlet allocation
NLP: natural language processing
PCA: principal component analysis
RQ: research question
S-BERT: Sentence Bidirectional Encoder from Transformers
TF-IDF: term frequency inverse document frequency
VADER: Valence Aware Dictionary and Sentiment Reasoner
